# Uptake of HIV testing in Burkina Faso: an assessment of individual and community-level determinants

**DOI:** 10.1186/s12889-017-4417-2

**Published:** 2017-05-22

**Authors:** Fati Kirakoya-Samadoulougou, Kévin Jean, Mathieu Maheu-Giroux

**Affiliations:** 1 Centre de Recherche en Epidémiologie, Biostatistiques et Recherche Clinique, École de Santé Publique, Université Libre de Bruxelles, Brussels, Belgium; 2 Department of Infectious Disease Epidemiology, Imperial College London, St Mary’s Hospital, London, UK; 3 Laboratoire MESuRS (EA 4628), Conservatoire National des Arts et Métiers, Paris, France; 4 Conservatoire National des Arts et Métiers, Unité PACRI, Institut Pasteur, Paris, France; 5 Department of Epidemiology, Biostatistics, and Occupational Health, McGill University, Montréal, Canada

**Keywords:** HIV/AIDS, Contextual determinants, Human immunodeficiency virus, Multilevel models, Voluntary counselling and testing, West Africa

## Abstract

**Background:**

Previous studies have highlighted a range of individual determinants associated with HIV testing but few have assessed the role of contextual factors. The objective of this paper is to examine the influence of both individual and community-level determinants of HIV testing uptake in Burkina Faso.

**Methods:**

Using nationally representative cross-sectional data from the 2010 Demographic and Health Survey, the determinants of lifetime HIV testing were examined for sexually active women (*n* = 14,656) and men (*n* = 5680) using modified Poisson regression models.

**Results:**

One third of women (36%; 95% Confidence Interval (CI): 33–37%) reported having ever been tested for HIV compared to a quarter of men (26%; 95% CI: 24–27%). For both genders, age, education, religious affiliation, household wealth, employment, media exposure, sexual behaviors, and HIV knowledge were associated with HIV testing. After adjustment, women living in communities where the following characteristics were higher than the median were more likely to report uptake of HIV testing: knowledge of where to access testing (Prevalence Ratio [PR] = 1.41; 95% CI: 1.34–1.48), willing to buy food from an infected vendor (PR = 2.06; 95% CI: 1.31–3.24), highest wealth quintiles (PR = 1.18; 95% CI: 1.10–1.27), not working year-round (PR = 0.90; 95% CI: 0.84–0.96), and high media exposure (PR = 1.11; 95% CI: 1.03–1.19). Men living in communities where the proportion of respondents were more educated (PR = 1.23; 95% CI: 1.07–1.41) than the median were more likely to be tested.

**Conclusions:**

This study shed light on potential mechanisms through which HIV testing could be increased in Burkina Faso. Both individual and contextual factors should be considered to design effective strategies for scaling-up HIV testing.

## Background

HIV testing uptake is a key component of UNAIDS’ newly adopted strategic framework. This framework calls for 90% of people living with HIV (PLWH) being aware of their status (diagnosed), 90% of those diagnosed receiving treatment, and 90% of those receiving treatment being virally suppressed by 2020 (i.e., the 90–90-90 objective) [1]. Voluntary counseling and testing (VCT) services for HIV represents the main entry point for prevention and care [2], and testing is considered one of the most cost-effective ways to decrease HIV transmission [3, 4]. However, across sub-Saharan African countries, high proportions of PLWH are still unaware of their status, with large within-country variations [5]. Important HIV testing scale-up efforts, and subsequent rapid linkage to care [6], are thus needed to reach UNAIDS’ objectives [7].

Expanding HIV testing requires a fine understanding of the individual and contextual variables that can act as barriers or facilitators to its uptake. Some individual predictors of HIV testing uptake, including wealth and education, have been consistently identified across sub-Saharan Africa [8–15]. On the other hand, factors such as sexual behaviors, and knowledge and attitudes toward HIV/AIDS have been found to be more variable across gender, as well as social and epidemiological settings [9, 16–21]. The health of individuals and their behaviors are shaped not only by individual factors, but also by the social environment’s structure in which they live [22, 23]. An analysis of the importance of contextual factors on health outcomes could allow for a better understanding of the complex linkage between individual and contextual determinants of HIV testing uptake and, ultimately, lead to innovative community level interventions [24]. A previous study from eight African countries (not including Burkina Faso) highlighted the association between HIV testing uptake and community-level demographic, economic, and behavioral determinants [25]. However, the study only examined testing uptake among married men and efforts to assess community-level predictors of HIV testing have been limited [18].

HIV seroprevalence in Burkina Faso was estimated in 2010 at 1.2% and 0.8% among women and men, respectively [26]. Substantial regional variations were observed, ranging from 0.2% in the Plateau Central to 2.0% in Central. Nearly 60% of PLWH in Burkina Faso reported having never been tested for HIV [26], highlighting an important gap to reach UNAIDS’s targets. The government of Burkina Faso - with international donors and many non-governmental organizations – have recently introduced and implemented programs to increase testing [27]. In this context, improving our understanding of individual and contextual barriers to HIV testing could contribute to its scale-up.

This study aims to examine individual and community determinants associated with lifetime HIV testing in Burkina Faso using a nationally representative sample of Burkinabè men and women. Understanding HIV testing uptake in relation to community characteristics, over and above individual factors, may provide new insights into the dynamics of HIV testing and help the national HIV program to more efficiently allocate their resources.

## Methods

### Data

This study uses data collected during the 2010 Burkina Faso Demographic and Health Survey (DHS), the first nationally-representative survey in Burkina Faso to collect information on previous HIV testing among both women and men. The survey protocol has been published elsewhere [26]. Briefly, the survey used a stratified, two-stage cluster sampling design. The country was stratified into rural and urban areas. Among each stratum, the first stage consisted of a random sample of clusters (primary sampling unit, PSU) using sampling probabilities proportional to the number of households in the cluster. Clusters were established by a general population and housing census conducted in 1996 (the average cluster populations were 1000 and 1200 in rural and urban areas, respectively). The second stage involved the systematic sampling of households from the selected clusters. A nationally representative sample of 14,957 households was thus obtained. All women aged 15 to 49 years were eligible to be interviewed. For men, a sub-sample of half of the selected households was randomly selected, from which all men aged 15 to 59 were eligible to be interviewed. Hereafter, the term community refers to all respondents living within the same geographical environment, corresponding to the PSU of the survey.

### Measures

The main outcome for this study was the participants’ self-reports of having ever been tested for HIV. Our independent variables were individual-level or community-level characteristics.

Individual variables covering socio-demographical, economical and behavioral dimensions, as well as knowledge and attitudes toward HIV/AIDS, were considered in the analyses. Socio-demographic covariates included: age, education, marital status, religion, occupational status, media exposure, location of residence (urban/rural), and an asset-based index of wealth [28]. The number of children ever born was also included for women. Sexual behavior was measured by self-reported lifetime number of sexual partners. HIV/AIDS knowledge was assessed by creating an index of correct responses to five (for men) and nine (for women) questions related to HIV transmission. Those sets of questions were summarized using principal component analysis, where the first axis explained 34% and 32% of the variance for women and men, respectively. This first axis was used to create a three-category index of HIV knowledge. Stigma towards PLWH was proxied using a set of four questions included in the DHS questionnaires: 1) willingness to share a relative’s HIV infection status, 2) willingness to care for an infected relative, 3) belief that a female teacher infected with HIV should teach, and 4) willingness to buy food from an HIV infected vendor. This choice of independent variables was guided by prior research on HIV testing in sub-Saharan Africa countries [18, 25].

The main explanatory community-level variables of interest were: HIV prevalence, knowledge of HIV testing service, HIV/AIDS knowledge, HIV-related stigma (percentage of respondents with accepting attitudes towards people living with AIDS), and community socio-demographic characteristics (proportion of respondents who are at least high-school educated in the community, proportion of households belonging to the poorest quintiles of wealth, and proportion of individuals reporting low media exposure). Consideration of these variables was based on their potential to be modified and their known or hypothesized association with HIV testing [22, 25].

Community-level variables were aggregated from individual responses pertaining to individuals of the same sex and the same PSU (except for HIV prevalence which was aggregated at the regional level due to low number of cases). To avoid endogeneity problems related to the double inclusion of variables in the model – first at the individual-level and again at the aggregate-level – community-level variables were calculated by excluding an individual’s own response from the numerator and denominator of the aggregate-level variables. Indicators from each of the community-level determinants of HIV testing uptake were dichotomized using the median as a cut-off point. The stigma question regarding willingness to care for an infected relative was not considered at the community level because preliminary analyses showed that, for both women and men, the communities had low levels of stigma with small between-community variations that were not deemed qualitatively important.

### Statistical analysis

For descriptive statistics at the individual and community levels, estimates accounted for the complex survey design using the sampling weights provided by DHS [29]. Prevalence ratios (PR) were obtained using a modified Poisson regression model that used *Generalized Estimating Equation* to take into account clustering of observations and perform the unbiased variance estimation [30, 31]. We did not adjust for survey weighting in the regressions. Univariate and multivariable analyses of individual-level covariates were first conducted. A complete case analysis was used and missing observations for women (*n* = 385) and men (*n* = 100) were disregarded. This was followed by multilevel models integrating the community-level variables. All multilevel models were adjusted for the following individual-level covariates: age, education, number of children ever born (for women only), marital status, religion, wealth index, place of residence, media exposure, HIV knowledge, lifetime number of sexual partners, and the four personal stigma questions. Two multilevel models were fitted for both gender. The first multilevel model adjusted for individual-level variables and each of the main community-level determinants hypothesized to be related to the community variation in HIV testing separately: 2010 HIV prevalence, knowledge about VCT service, HIV/AIDS knowledge and HIV related stigma (model 1). The second multilevel model is fully adjusted for all individual-level and community-level variables enumerated above (model 2). Analyses were restricted to participants reporting having ever had sexual intercourse. All analyses were stratified by gender because attitudes toward HIV testing are likely to vary between women and men [32, 33]. Statistical analyses were performed using the R statistical software and the “*geepack*” package was used to fit the regression models [34].

## Results

The sample of the 2010 Burkina Faso DHS consisted of 14,536 households, among 14,957 identified ones (421 households were unoccupied), and 17,087 women and 7307 men completed interviews. The participants’ response rate was 98.4% for women and 97.3% for men. Inferences are thus based on the analyses of 14,373 women and 5680 men who reported having had sexual intercourse and without missing observations.

### Characteristics of the study population

The sociodemographic characteristics of the study population are presented in Table 1. About half of the male participants were aged 35 years or older whereas only a third of female respondents were in this age group. About two thirds of respondents had no schooling and pproximatively 90% of women and 80% of men were married or living in a union. More than half were Muslims and two thirds lived in rural areas.

**Table 1:** Socio-demographic characteristics of women and men participated in the Burkina Faso 2010 Demographic and Health Survey

Variables		Women		Men	
		*N*	Proportion (%)^a^	*N*	Proportion (%)^b^
Age groups					
	15–24	4403	30.3	1072	18.4
	25–34	5441	36.9	1791	31.4
	≥35	4914	32.9	2917	50.2
Education					
	No school	11,393	78.0	3722	65.6
	Primary	1877	12.2	1076	18.5
	Secondary/higher	1488	9.9	982	15.9
Number of children ever born					
	0	1655	11.3		
	1–2	4052	27.3		
	3+	9051	61.4		
Marital status					
	Married/in union	13,236	90.6	4528	79.5
	Single	1522	9.4	1252	20.5
Religion					
	Muslim	8961	62.5	3458	61.0
	Christian	4330	28.6	1681	28.5
	Animist/others	1426	8.6	635	10.4
	*Missing*	*41*	*0.2*	*6*	*0.1*
Wealth index					
	Poorest	2475	17.7	940	17.1
	Poor	2747	19.3	1058	19.0
	Middle	2900	19.6	1032	18.1
	Richer	3160	20.4	1182	19.3
	Richest	3476	23.1	1568	26.4
Place of residence					
	Urban	4385	25.2	1967	29.2
	Rural	10,373	74.8	3813	70.8
Working year-round					
	Yes	3081	20.0	2133	36.8
	No	11,632	79.7	3633	62.9
	*Missing*	*45*	*0.3*	*14*	*0.3*
Media exposure^b^					
	Low (0–1)	6165	42.2	998	18.3
	Middle (2–3)	6271	42.1	3053	53.3
	High (4–6)	2258	15.4	1715	28.2
	*Missing*	*64*	*0.4*	*14*	*0.2*

^a^Proportions take into account sample weights

^b^Exposure to mass media was measured through a composite index of three survey items that assessed whether the respondent reads newspapers or magazines, listens to the radio, or watches television. The additive scale is split into a three-level categorical variable: low media exposure (score of 0–1), medium media exposure (2–3), and high media exposure (4–6)

Among men and women, three-quarters of participants knew where they could get an HIV test (Table 2). Men were more educated, had higher level of media exposure, and higher HIV/AIDS knowledge than women. Additionally, men were more likely to have views that stigmatized HIV/AIDS than women (21% vs. 9% expressed stigmatizing views to all four stigma-related questions) but few individuals had no stigmatizing views on all questions (7% and 6% of women and men, respectively).

**Table 2:** Knowledge of access to HIV testing, HIV knowledge, sex behaviors, and HIV stigma in women and men of Burkina Faso, 2010

Variables		Women		Men	
	*N*	Proportion (%)^a^	*N*	Proportion (%)^a^	
Access/HIV knowledge^**b**^					
Know a place to get tested	No	3345	24.3	1295	24.6
	Yes	11,400	75.6	4481	75.3
	Missing	13	0.1	4	0.1
HIV knowledge score	Low	4888	34.2	1731	31.8
	Medium	4881	34.6	1588	26.8
	High	4867	30.4	2440	40.9
	*Missing*	*122*	*0.8*	*21*	*0.4*
Sexual behavior					
Lifetime number of sexual partners	1	11,026	75.4	1169	21.4
	2	2812	18.7	1454	26.0
	3 or more	904	5.8	3121	51.9
	*Missing*	*16*	*0.1*	*36*	*0.7*
Personal stigma					
If a relative would become HIV positive, willing to share his/her infection status	No (stigma)	10,894	73.9	3193	57.6
	Yes	3860	26.1	2587	42.4
	*Missing*	*4*	*0.0*	*0*	*0.0*
Willing to care for an HIV positive relative in their own house	No (stigma)	2314	16.5	473	9.3
	Yes	12,443	83.5	5307	90.7
	*Missing*	*1*	*0.0*	*0*	*0.0*
Believes that an HIV positive female teacher (without symptoms) should teach	No (stigma)	5466	38.9	1917	35.4
	Yes	9292	61.1	3862	64.4
	*Missing*	*0*	*0.0*	*1*	*0.0*
Willing to buy food from an HIV positive vendor	No (stigma)	9402	64.8	3078	56.2
	Yes	5355	35.2	2694	43.6
	*Missing*	*1*	*0.0*	*8*	*0.1*

^a^Proportions take into account sampling weights

^b^The five (men) and nine (women) questions entering the HIV knowledge index are: people can protect themselves from contracting HIV by (1) using condoms; (2) having sex only with one faithful, uninfected partner; (3) people knowing that mosquitoes can’t transmit HIV and (4) that it cannot be transmitted by sharing food with an HIV-infected person; (5) a healthy looking person can have the AIDS virus; and, for women only, (6) people who report that HIV can be transmitted from mother to child during pregnancy, (7) delivery, and (8) through breastfeeding; and (9) know drugs to avoid AIDS transmission to baby during delivery and breastfeeding

### HIV testing uptake

**Fig. 1:**
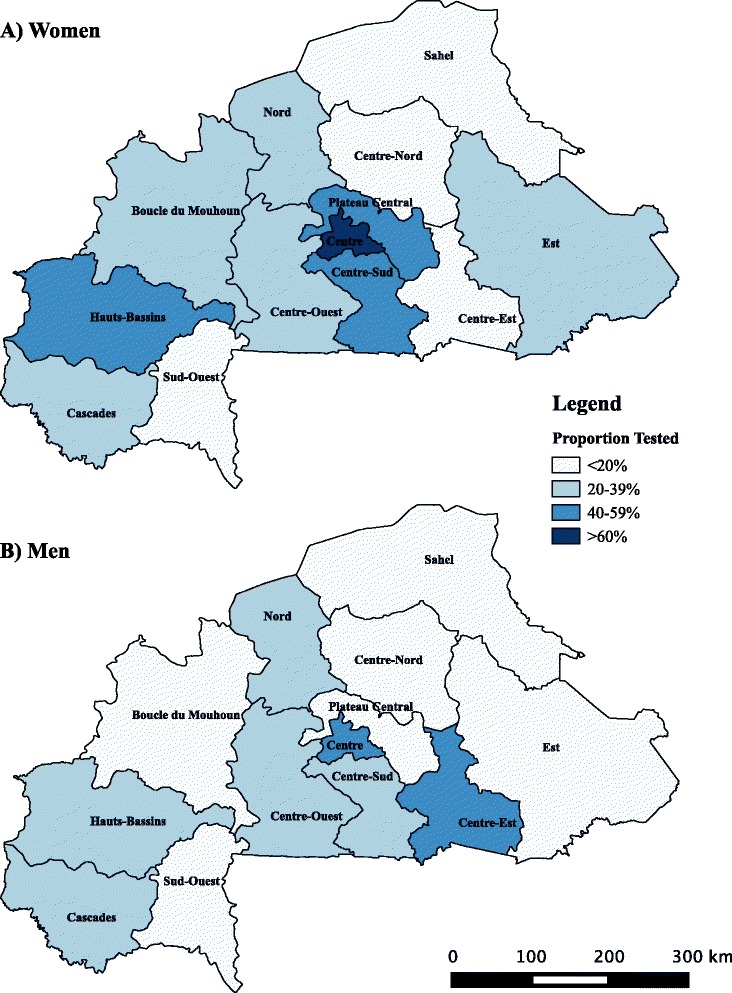
Uptake of HIV testing in Burkina Faso. Proportion of **a** women and **b** men that reported having ever been tested for HIV in the country’s 13 administrative regions in 2010. (Map is our own)

The proportion of women reporting having ever been tested for HIV was 35.6% (95% Confidence Interval [CI]: 32.9–37.4%) while this proportion was 10% lower for men (25.6%; 95% CI: 24.2–26.6%). Among women having ever been tested, 90.8% have had an HIV test as part of an antenatal care visit. HIV testing differed greatly geographically and was highest for both genders in the department of *Central Region*, where the capital Ouagadougou is located, at 61.9% for women and 46.6% for men (Figs. 1a, b). Lowest uptake of HIV testing was found in in the *Sahel* for women (16.4%) and the *Boucle du Mouhoun* departments for men (13.9%).

### Individual-level determinants of HIV testing uptake

For both genders, uptake of HIV testing was associated with age, education, religious affiliation, household wealth index, being employed year-round, media exposure, number of lifetime sexual partners, and level of HIV knowledge (Table 3). The probability of having been tested was highest among women aged 15–24 and men aged 25–34 years. Having non-stigmatizing views on PLWH was associated with HIV testing uptake but this was not consistent for all stigma-related questions. For women, the number of children ever born was significantly associated with HIV testing.

**Table 3:** Univariate and multivariable analyses of individual-level determinants of HIV testing uptake, stratified by gender

Variables		Women (*N* = 14,373)		Men (*N* = 5680)	
		Univariate	Multivariable	Univariate	Multivariable
		PR^a^ (95% CI)	PR^a^ (95% CI)	PR^a^ (95% CI)	PR^a^ (95% CI)
Socio-demographic					
Age groups					
	15–24	Referent	Referent	Referent	Referent
	25–34	**1.06 (1.01–1.11)**	**0.87 (0.83–0.92)**	**1.21 (1.07–1.35)**	**1.20 (1.06–1. 35)**
	>35	**0.69 (0.65–0.74)**	**0.61 (0.57–0.66)**	**0.80 (0.71–0.90)**	0.99 (0.86–1.14)
Education					
	No school	Referent	Referent	Referent	Referent
	Primary	**1.80 (1.70–1.90)**	**1.16 (1.10–1.23)**	**2.22 (1.96–2.51)**	**1. 35 (1.18–1.54)**
	Secondary/higher	**2.53 (2.42–2.65)**	**1.29 (1.21–1.37)**	**4.83 (4.39–5.31)**	**2. 01 (1.76–2.30)**
Number of children ever born					
	0	Referent	Referent	Not applicable	
	1–2	**1.33 (1.23–1.43)**	**1.63 (1.51–1.75)**		
	3+	0.93 (0.86–1.00)	**1.78 (1.63–1.94)**		
Marital status					
	In Union	Referent	Referent	Referent	Referent
	Single	**1.24 (1.17–1.32)**	0.95 (0.89–1.01)	**1.35 (1.23–1.49)**	0.95 (0.86–1.06)
Religion					
	Muslim	Referent	Referent	Referent	Referent
	Christian	**1.28 (1.22–1.34)**	**1.09 (1.05–1.14)**	**1.49 (1.36–1.62)**	**1.11 (1.03–1.20)**
	Animist/others	**0.72 (0.64–0.80)**	0.99 (0.89–1.11)	**0.49 (0.38–0.62)**	**0.79 (0.63–1.00)**
Wealth index					
	Poorest	Referent	Referent	Referent	Referent
	Poorer	**1.33 (1.19–1.48)**	**1.26 (1.13–1.40)**	**1.37 (1.04–1.81)**	1.12 (0.85–1.47)
	Middle	**1.48 (1.33–1.64)**	**1.30 (1.18–1.44)**	**2.02 (1.56–2.62)**	**1.50 (1.16–1.94)**
	Richer	**1.88 (1.70–2.08)**	**1.44 (1.31–1.59)**	**3.23 (2.54–4.12)**	**1.91 (1.48–2.46)**
	Richest	**3.28 (3.00–3.60)**	**1.57 (1.41–1.76)**	**6.69 (5.33–8.40)**	**1.90 (1.45–2.49)**
Place of residence					
	Urban	Referent	Referent	Referent	Referent
	Rural	**0.44 (0.42–0.46)**	**0.75 (0.71–0.80)**	**0.34 (0.31–0.37)**	0.90 (0.80–1.01)
Working year-round					
	Yes	Referent	Referent	Referent	Referent
	No	**0.62 (0.59–0.65)**	**0.90 (0.86–0.94)**	**0.48 (0.44–0.53)**	**0.86 (0.79–0.94)**
Media exposure					
	Low	Referent	Referent	Referent	Referent
	Middle	**1.39 (1.31–1.47)**	**1.13 (1.07–1.20)**	**1.66 (1.37–2.03)**	**1.23 (1.01–1.49)**
	High	**2.52 (2.38–2.67)**	**1.21 (1.13–1.30)**	**4.9 (4.06–5.93)**	**1.49 (1.20–1.84)**
HIV knowledge					
	Low (1–3)	Referent	Referent	Referent	Referent
	Medium (4–5)	**1.56 (1.46–1.66)**	**1.31 (1.23–1.39)**	**1.55 (1.32–1.82)**	**1.18 (1.00–1.38)**
	High (6–7)	**2.10 (1.98–2.23)**	**1.45 (1.37–1.54)**	**3.22 (2.81–3.68)**	**1.29 (1.12–1.49)**
Sexual behavior					
Lifetime sexual partners					
	1	Referent	Referent	Referent	Referent
	2	**1.25 (1.19–1.32)**	**1.09 (1.04–1.14)**	1.12 (0.96–1.31)	1.06 (0.93–1.22)
	3 and +	**1.71 (1.60–1.82)**	**1.18 (1.11–1.25)**	**1.59 (1.40–1.80)**	**1.22 (1.08–1.37)**
Stigma-related questions					
Willing to share a relative’s HIV infection status					
	No (stigma)	Referent	Referent	Referent	Referent
	Yes	**0.94 (0.89–0.99)**	**0.93 (0.89–0.98)**	**1.33 (1.22–1.45)**	1.06 (0.98–1.14)
Willing to care for an infected relative					
	No (stigma)	Referent	Referent	Referent	Referent
	Yes	**1.94 (1.78–2.12)**	**1.24 (1.14–1.36)**	**2.68 (2.06–3.48)**	0.99 (0.73–1.34)
Believe female teacher infected with HIV should teach					
	No (stigma)	Referent	Referent	Referent	Referent
	Yes	**1.91 (1.80–2.01)**	**1.16 (1.09–1.24)**	**2.97 (2.60–3.40)**	**1.32 (1.14–1.54)**
Willing to buy food from an infected vendor					
	No (stigma)	Referent	Referent	Referent	Referent
	Yes	**2.13 (2.04–2.23)**	**1.36 (1.29–1.43)**	**2.97 (2.68–3.29)**	**1.38 (1.23–1.56)**

Statistically significant results are bolded.

^a^Prevalence Ratios (95% Confidence Interval)

### Community-level determinants of HIV testing uptake

Table 4 presents results of the community-level determinants of HIV testing. For model 1, community-level determinants are adjusted only for selected individual-level variables. On the other hand, model 2 is fully adjusted for individual and all community-level determinants listed in Table 4. Results from this fully adjusted model (model 2) suggested that women were significantly more likely to report testing in communities where a larger percentage of respondents knew the location of a VCT service, where views towards buying food from HIV positive vendors were less stigmatizing, where the proportion of respondents in the highest wealth quintile was higher, and in communities with the highest media exposure. Furthermore, women residing in community where the proportion of respondents not working year-round was higher than the median were 10% less likely to report having ever been tested. For men, few community determinants reached statistical significance for both model 1 and 2. Men were more likely to report previous testing in communities where respondents were more educated.

**Table 4:** Multivariable analysis of community-level determinants of HIV testing uptake in Burkina Faso women and men

Variable0073	Women (*N* = 14,373)		Men (*N* = 5680)	
	Model 1^a^	Model 2^a^	Model 1^a^	Model 2^a^
	PR (95% CI)	PR (95% CI)	PR (95% CI)	PR (95% CI)
Community with higher HIV prevalence	0.97 (0.93–1.02)	1.00 (0.96–1.05)	1.06 (0.97–1.16)	1.06 (0.97–1.16)
Community with higher knowledge of place to get tested	**1.43 (1.36–1.50)**	**1.41 (1.34–1.48)**	1.01 (0.92–1.10)	1.01 (0.93–1.11)
Community with lower HIV/AIDS knowledge	0.96 (0.91–1.00)	1.01 (0.96–1.06)	1.03 (0.94–1.13)	1.04 (0.95–1.14)
Community more willing to share a relative’s infection HIV status	1.02 (0.98–1.06)	1.01 (0.97–1.06)	0.97 (0.89–1.05)	0.97 (0.89–1.06)
Community believing female teacher infected with HIV should teach	**1.18 (1.07–1.30)**	1.01 (0.92–1.12)	0.84 (0.68–1.04)	0.81 (0.64–1.04)
Community more willing to buy food from an infected vendor	**2.17 (1.39–3.36)**	**2.06 (1.31–3.24)**	0.83 (0.55–1.24)	0.91 (0.57–1.43)
Community with more educated respondents	**1.17 (1.10–1.25)**	1.01 (0.94–1.08)	**1.24 (1.09–1.41)**	**1.23 (1.07–1.41)**
Community with higher proportion of respondents not working year-round	**0.82 (0.77–0.87)**	**0.90 (0.84–0.96)**	0.90 (0.79–1.03)	0.95 (0.82–1.10)
Community with more respondents in the highest wealth quintile	**1.29 (1.21–1.38)**	**1.18 (1.10–1.27)**	1.10 (0.95–1.26)	1.00 (0.85–1.17)
Community with higher media exposure	**1.22 (1.15–1.30)**	**1.11 (1.03–1.19)**	1.12 (0.97–1.28)	1.06 (0.91–1.22)

Statistically significant results are bolded

*PR* Prevalence Ratio, *95*% *CI* 95% Confidence Interval

^a^Model 1 has each community variable included separately in the model and adjusted for the following individual-level determinants: age, education, number of children ever born (for women only), marital status, religion, wealth index, place of residence, media exposure, HIV knowledge, lifetime number of sexual partners, and personal stigma. Model 2 is fully adjusted for all variables listed in the table and the individual-level determinants adjusted for in Model 1

## Discussion

Using nationally representative data of sexually active women and men from Burkina Faso, we observed low levels of HIV testing uptake in 2010 with only a third of women and a quarter of men reporting having ever been tested for HIV. We identified several determinants of HIV testing uptake acting at individual and community levels, and their effect often varied by gender. Individual factors associated with HIV testing uptake included socio-demographic, economic, behavioral factors as well as knowledge and attitudes related to HIV. Community-level variables associated with HIV testing uptake were mostly related to education, wealth, occupational status, media exposure, and stigma.

Our study identified various individual correlates of HIV testing uptake. As previously documented, we observed among both men and women that testing uptake increased with educational level and wealth [13, 25, 35, 36]. Similarly, high HIV-related knowledge and access to broader information channels, through media exposure, was associated with HIV testing [35–39]. These results highlight the importance of providing health education to both women and men while deploying targeted efforts to reach populations with low uptake of HIV testing. Some of the barriers to HIV testing uptake that we identified in this study may be related to the HIV testing offer modalities, however. For example, lower testing levels among participants living in rural areas may be related to lower accessibility of HIV testing sites in these regions.

The present study expands upon previous literature by providing evidence of gendered patterns of association between community determinants and HIV testing. Our results suggest that tested women were more likely to live in communities with high access to testing resources/facilities, where women are wealthier, and with better media exposure. For men, uptake of VCT was higher for those living in communities with larger proportions of respondents with secondary/higher education.

We observed that uptake of VCT was generally higher among individuals with less stigmatizing views. Decision-making about HIV testing are often linked to an individual’s social network influences and addressing HIV-related stigma could improve community norms about testing. The recent HPTN-043 ACCEPT cluster-randomized trial demonstrated how addressing HIV-related community norms may translate into lower HIV-related stigma and increase uptake of HIV testing [40, 41]. This is especially relevant for Burkina Faso where levels of stigma were found to be high: only 7% of women and 5% of men expressed no stigmatizing attitudes about PLWH.

We acknowledge some limitations in this study. The measures used in this paper were self-reported and therefore susceptible to social desirability biases. The use of cross-sectional data also makes the temporal sequences between some covariates and testing uptake in this study unknowns. Furthermore, communities were defined based on PSU memberships. Because social networks do not necessarily follow the dimensions of a PSU, we might have under or overestimated effect size measures of the community-level determinants. Additionally, we were not able to account for the availability and proximity of health infrastructures, as well as the quality of their health services. Finally, female sex workers and men who have sex with men, key populations at high risk of HIV acquisition and transmission, could be underrepresented in population-based household surveys such as this one.

Strengths of this study included its large sample size and high response rate. To our knowledge, this study is the first to identify correlates of HIV testing uptake among a nationally-representative sample of the Burkinabè population. Furthermore, by aggregating data at the PSU level we examined community-level determinants while avoiding issues of ecological fallacy.

## Conclusions

To reach UNAIDS’ 90–90-90 target, HIV testing in Burkina Faso should be considerably scaled-up in the coming years. Among individuals who were found to be HIV positive in the 2010 seroprevalence survey, about 60% reported having never been tested for HIV. This points to a potentially important gap in the national response. Given that more than 50% of new HIV infection in Burkina Faso occur among stable couples [42], interventions to scale-up testing will need to focus on these partnerships. HIV testing interventions among members of key populations, such as female sex workers and men who have sex with men, also warrants further consideration [43–46]. To increase HIV testing uptake, several interventions and policies could be considered. First, accessibility to HIV diagnosis should be improved in rural areas with high HIV prevalence. Testing campaigns in particular have been shown to reach high population coverage and uptake. Furthermore, mobile HIV testing campaigns may be particularly effective in increasing HIV testing coverage in rural settings, among men, and young adults [47, 48]. Socio-economic inequalities in HIV testing could be reduced by the implementation of specific testing modalities such as the systematic proposition of an HIV test by health care workers [12]. However, restricting the offer of HIV testing to health facilities would not be sufficient to increase HIV testing among populations having few contacts with healthcare providers, especially the adult male population [48]. Attempts to offer HIV testing outside health facilities may thus be an effective way to increase uptake [49]. Especially, self-testing for HIV may be a relevant option to be considered in contexts of high HIV-related stigma [50].

Reaching the undiagnosed PLWH in a timely manner is a crucial and necessary step for individuals to benefit from antiretroviral treatment and to sustainably reduce population-level HIV transmission [51, 52]. Implementing effective policies to address individual and community-level barriers to testing is required to achieve this objective.

## Abbreviations

CI: Confidence intervals
DHS: Demographic and Health Survey
HIV: Human immunodeficiency virus
PLWH: People living with HIV
PR: Prevalence ratios
PSU: Primary sampling unit
VCT: Voluntary counselling and testing
